# Evaluation of Risk Score for Isolated Surgical Aortic Valve Replacement and Transcatheter Aortic Valve Replacement—Results from the German National Quality Database

**DOI:** 10.1093/icvts/ivaf307

**Published:** 2025-12-22

**Authors:** Andreas Böning, Andreas Beckmann, Markus Heinemann, Torsten Doenst, Zulfugar T Taghiyev, Bernd Niemann

**Affiliations:** Department of Cardiovascular Surgery, Justus Liebig University, Rud.-Buchheim-Str. 7, Giessen 35385, Germany; German Society for Thoracic, Cardiac and Vascular Surgery, Berlin, Luisenstraße 58/59, Berlin 10117, Germany; German Society for Thoracic, Cardiac and Vascular Surgery, Berlin, Luisenstraße 58/59, Berlin 10117, Germany; Clinic for Cardiac Surgery and Pediatric Cardiac Surgery, Heart Center Duisburg, Fahrner Str. 133, Duisburg 47169, Germany; German Society for Thoracic, Cardiac and Vascular Surgery, Berlin, Luisenstraße 58/59, Berlin 10117, Germany; German Society for Thoracic, Cardiac and Vascular Surgery, Berlin, Luisenstraße 58/59, Berlin 10117, Germany; Department of Cardiac and Thoracic Surgery, University Hospital Jena, Am Klinikum 1, Jena 07747, Germany; Department of Cardiovascular Surgery, Justus Liebig University, Rud.-Buchheim-Str. 7, Giessen 35385, Germany; Department of Cardiovascular Surgery, Justus Liebig University, Rud.-Buchheim-Str. 7, Giessen 35385, Germany

**Keywords:** surgical aortic valve replacement, transcatheter aortic valve replacement, TAVI, SAVR

## Abstract

**Objectives:**

For the treatment of aortic valve stenoses, both surgical aortic valve replacement (SAVR) and transcatheter aortic valve implantation (TAVI) are available. We compared the frequently used Euroscore with the AKL-Cath- and the AKL-Chir Score, describing the mortality risk of the 2 different treatment methods.

**Methods:**

Based on a retrospective cohort study using mandatory quality assurance data, we analysed the frequency and the outcomes (primary end-point: in-hospital mortality) of all patients treated in Germany between 2015 and 2020. The observed results were compared to the predicted risk using the Euroscore, the AKL-Cath Score, and the AKL-Chir Score.

**Results:**

Our data show a reduction in the number of isolated SAVR procedures from 9790 in 2015 to 6106 in 2020, corresponding to a 37.6% decrease. Over the same period, the number of TAVI procedures increased from 15 653 to 21 501, an increase of 37.3%. Regarding in-hospital mortality following TAVI, there was a decline from 4% (2015) to 2.5% (2020), while in-hospital mortality following SAVR remained nearly constant at 3%. Over the study period, there is an overestimation of TAVI risk while simultaneously underestimating SAVR risk by EuroSCORE II. In contrast, the mortality risk of patients is well estimated using the AKL-Kath Score in the TAVI group and the AKL-Chir Score in the SAVR group. The AKL-Chir Score in TAVI patients overestimates their mortality, while the AKL-Kath Score underestimates the mortality of SAVR patients.

**Conclusions:**

AKL-Chir score and AKL-Cath score estimate the mortality risk of SAVR and TAVI patients more precisely than the Euroscore II.

## Graphical abstract

**Figure:**
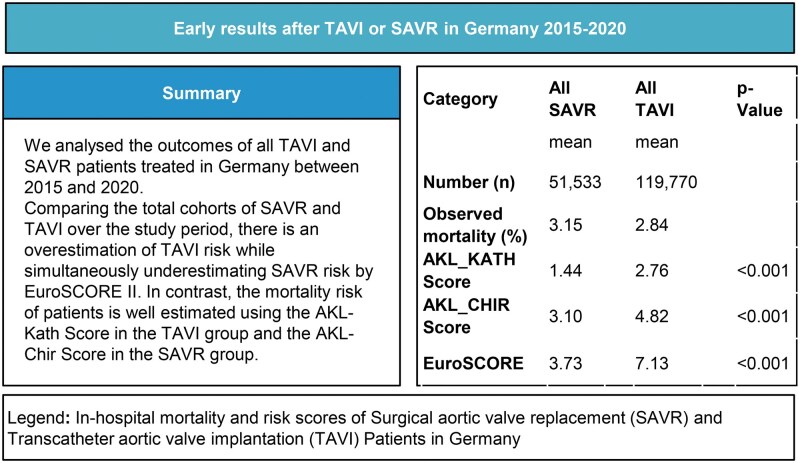

## INTRODUCTION

The invasive treatment of aortic valve diseases comprises 2 different methods: isolated surgical aortic valve replacement (SAVR) under general anaesthesia with the use of cardiopulmonary bypass (CPB) or transcatheter aortic valve implantation (TAVI), usually under conscious sedation and without CPB on the beating heart. According to the European guidelines for the “Management of Valvular Heart Disease 2021” by the European Association of Cardiothoracic Surgery (EACTS) and the European Society of Cardiology (ESC), after evaluation by a heart team, SAVR is primarily recommended for patients with aortic stenosis who are younger than 75 years with a low surgical risk, whereas TAVI is recommended for those aged 75 or older with a high surgical risk.^1^ In Germany, approximately three-quarters of all patients with aortic stenosis are treated with TAVI.^2^ For patients with isolated aortic valve insufficiency and/or endocarditis, surgical SAVR is the treatment of choice according to specific criteria, while TAVI may only be considered for selected inoperable patients.^1^

In Germany, mandatory inpatient quality assurance has been legally regulated and firmly established for decades under the responsibility of the Federal Joint Committee (G-BA). Based on specific G-BA guidelines, a longitudinal analysis of data is made possible, focusing on the measurement of healthcare quality. Since 2015, central data collection has been conducted by the Institute for Quality and Transparency in Healthcare (IQTIG), an independent scientific institution responsible for legally mandated quality assurance in German healthcare. For isolated SAVR and TAVI, quality assurance was governed by the “Directive on Data-Driven Cross-Institutional Quality Assurance (DeQS-RL)”. As a result, IQTIG holds a complete dataset of statutorily insured patients treated with SAVR or TAVI, including 1-year follow-up data since 2020. IQTIG provides all data collected through mandatory quality assurance measures under §136(1) Sentence 1 No. 1 SGB V for secondary scientific purposes.

In 2013, the German Aortic Valve Score was published by Kötting et al,^3^ which is the base for the AKL-Kath score and the AKL-Chir-Score. The AKL-Kath score predicts the perioperative mortality risk of TAVI patients, while the AKL-Chir score predicts the risk of SAVR patients. Every year, the parameters on which the risk calculation is based are published by the IQTIG.

In Germany, health insurance is obligatory for every citizen. Therefore, around 90% of Germans are members of public health insurance companies, while around 10% are privately insured. Data for the nationwide quality assurance program come only from public health insurance patients, but not from privately insured patients.

At the request of the DGTHG, IQTIG analysed the data of patients treated with SAVR and TAVI in Germany between 2015 and 2020 according to a pre-specified evaluation plan, addressing the following questions:

1. What are the trends in the numbers of both treatment procedures (isolated SAVR and isolated TAVI) from 2015 to 2020?
2. How do the risk profiles of patients undergoing both treatment procedures evolve over time?
3. How reliable are the scores AKL-Chir Score, AKL-Kath Score, EuroSCORE II in assessing the outcomes of both procedures?

## METHODS

### Inpatient cases, treatment modalities, and group classification

This study compares the IQTIG datasets of all isolated SAVR and TAVI patients from 2015 to 2020.

The primary end-point analysed was in-hospital mortality. Secondary end-points included stroke of any kind and new pacemaker or ICD implantation. The observed results were compared to the predicted risk using the Euroscore, the AKL-Cath Score, and the AKL-Chir Score.

### Ethical statement

This study used routine data from a national database, stored and managed in accordance with the German data protection law. In Germany, mandatory inpatient quality assurance has been legally regulated and the IQTIG, having evaluated the data for this article, has been responsible for the data collection. As these data do not contain patient information, written patient informed consent was not required.

### Statistics

Descriptive statistics serve to describe the (sub)samples and examine the structural similarity of individual groups. For this purpose, demographic variables and influencing factors were compared separately according to intervention types (TAVI/SAVR) and subpopulations.

Categorical and quantitatively discrete variables were described as absolute and relative frequencies, relative to the sample size. Characteristics described using frequencies were compared using the Chi-square test (significance level *α* = .05).

Continuous variables were described by sample size, number of missing values, mean and standard deviation, minimum, 25th percentile, median, 75th percentile, and maximum. These variables were compared between subpopulations using the Mann-Whitney *U*-test.

In addition to directly recorded characteristics, the risk (using the risk adjustment models EuroSCORE II, AKL-Chir, and AKL-Kath Score) of all treated patients in the individual subpopulations per recording year was analysed descriptively.

Absolute and relative frequencies in the respective risk classes and the national average expected mortality (mean) with standard deviation were calculated.

As a primary end-point, in-hospital mortality was analysed. All statistical tests were conducted without adjustment for multiple testing. Therefore, our analyses are only exploratory in nature.

## RESULTS

**Figure 1.**
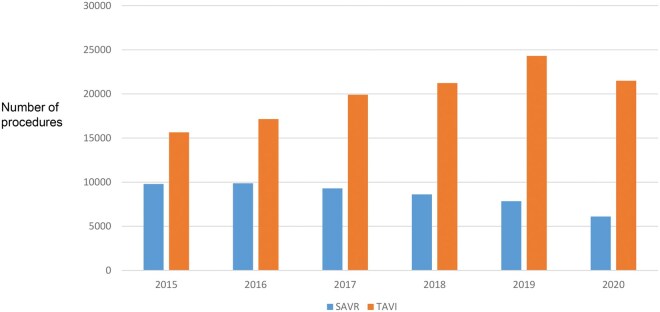
Number of Patients with TAVI and SAVR in Germany over Time

Between 2015 and 2020, 51 533 SAVR patients and 119 770 TAVI patients were included in the German mandatory quality assurance system. The data for this period show an annual reduction in isolated SAVR procedures from 9790 in 2015 to 6106 in 2020, a decrease of 37.6% (3684 patients). In contrast, TAVI procedures increased from 15 653 (2015) to 21 501 (2020), a 37.3% increase (5848). There was an absolute increase in aortic valve therapies over time of 8.5% from 25 443 to 27 607 (see **Figure 1**).

When comparing the demographic data (**Table 1**) of the SAVR group with the TAVI group, it is noticeable that the baseline characteristics of both the groups differ significantly in all aspects: TAVI patients are significantly older and have a higher prevalence of heart failure than SAVR patients. They also show significantly higher rates of atrial fibrillation, pacemaker implantations, coronary artery disease, prior surgeries, diabetes, peripheral artery disease, and lung diseases, which results in a significantly higher EuroSCORE (7.12 ± 7.73) compared to SAVR patients (3.73 ± 6.81).

**Table 1.** Preoperative Parameters of All SAVR Patients with All TAVI Patients from 2015 to 2020 (Ordinal Values)

Category	All SAVR (2015-2020)		All TAVI (2015-2020)		*P*-value
	*n*	%	*n*	%	
Number of patients	51 533		119 770		
BMI < 22	3902	7.57	13 345	11.14	<.001
NYHA = IV	3214	6.24	13 179	11.00	<.001
Myocardial infarction ≤ 48 h	194	0.38	840	0.70	<.001
Resuscitation ≤ 48 h	218	0.42	334	0.28	<.001
Pulmonary hypertension > 55 mmHg	1442	2.80	15 628	13.05	<.001
Atrial fibrillation	1219	2.37	3627	3.03	<.001
Pacemaker	1885	3.66	12 998	10.85	<.001
Defibrillator	500	0.97	2301	1.92	<.001
LVEF < 20%	678	1.32	2269	1.89	<.001
Coronary artery disease	11 902	23.10	69 108	57.70	<.001
Previous surgery	5465	10.60	20 068	16.76	<.001
Diabetes mellitus (insulin therapy)	4096	7.95	22 857	19.08	<.001
Peripheral arterial disease	2540	4.93	14 836	12.39	<.001
Carotid stenosis	2895	5.62	19 754	16.49	<.001
COPD	5219	10.13	14 460	12.07	<.001
Other lung diseases	2264	4.39	17 998	15.03	<.001
Preoperative stroke or cerebral haemorrhage	2977	5.78	6362	5.31	<.001
Acute renal replacement therapy	342	0.66	779	0.65	.755
Chronic renal replacement therapy	715	1.39	779	0.65	<.001
Catecholamine therapy	1146	2.22	5293	4.42	<.001

**Figure 2.**
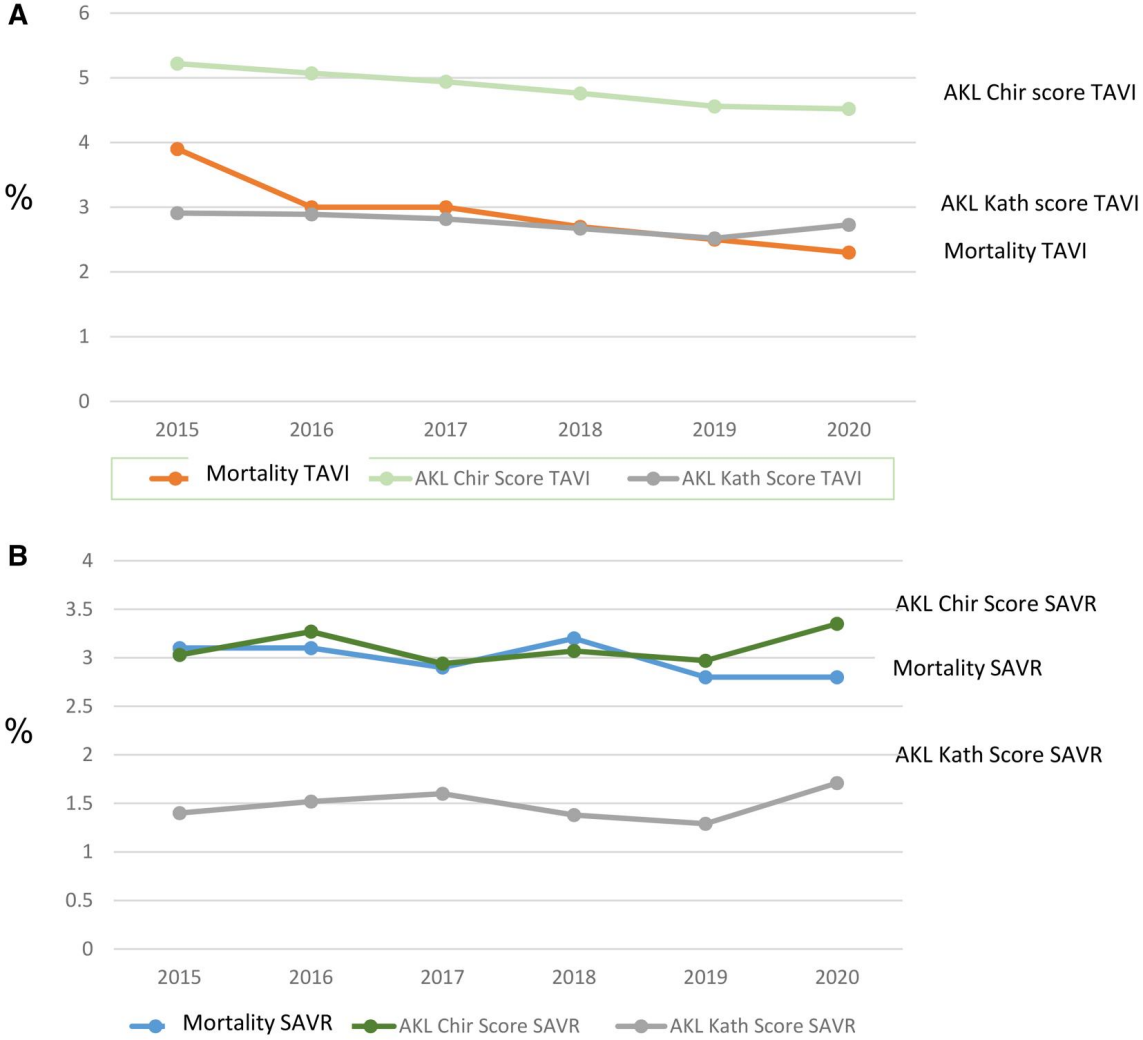
(A) Comparison between AKL-Chir score and AKL-Kath score in all SAVR patients. (B) Comparison between AKL-Chir score and AKL-Kath score in all TAVI patients

Over the study period, the risk profiles of patients in the SAVR and TAVI groups (overall) developed differently: While the median AKL-Kath Score and EuroSCORE II for TAVI patients steadily decreased from 2015 to 2019, the AKL-Chir Score and EuroSCORE II values for SAVR patients remained almost unchanged over the same period (see **Figures 1 and 2A and B**). In 2020, both the groups showed an increase in risk.

The mortality risk of patients is well estimated using the AKL-Kath Score in the TAVI group and the AKL-Chir Score in the SAVR group (see **Figure 2A and B**). In direct comparison, it is noticeable that applying the AKL-Chir Score to TAVI overestimates mortality, while using the AKL-Kath Score underestimates the mortality of SAVR patients.

**Figure 3.**
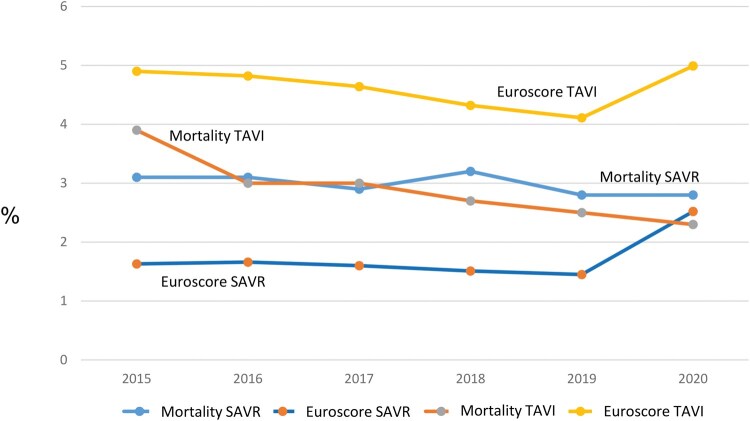
Comparison of Mortality in All SAVR and All TAVI Patients over the Entire Period (EuroSCORE II)

Comparing the total cohorts of SAVR and TAVI over the study period (**Table 2**), the ratio between the estimated mortality based on EuroSCORE II and the actual observed mortality is consistently negative for TAVI patients, whereas EuroSCORE II underestimates the observed mortality in the SAVR group (see **Figure 3**). There is an apparent systematic overestimation of TAVI risk while simultaneously underestimating SAVR risk by EuroSCORE II. Additionally, a decrease in mortality after TAVI is observed from 2015 (4%) to 2020 (2.5%), while it remains almost unchanged in the SAVR group at around 3%.

**Table 2.** In-Hospital Mortality and Risk Scores of SAVR Patients and TAVI Patients (Means and Standard Deviations)

Category	All SAVR		All TAVI		*P*-value
	Mean	±SD	Mean	±SD	
Number (*n*)	51 533		119 770		
Observed mortality (%)	3.15		2.84		
AKL_KATH_Score	1.44	3.12	2.76	3.46	<.001
AKL_CHIR_Score	3.10	6.70	4.82	5.52	<.001
EuroSCORE	3.73	6.81	7.13	7.73	<.001

While the rate of neurological complications was not significantly different (0.73% for SAVR vs 0.75% for TAVI), significantly fewer pacemakers were implanted perioperatively in the SAVR group (4.2%) compared to the TAVI group (11.7%).

## DISCUSSION

Firstly, our analysis of QA data from the 6-year period between 2015 and 2020 in Germany shows a continuous decline in SAVR procedures by 37.6%. During the same period, TAVI procedures increased by 37.3% (**Figure 1**). By 2020, 81% of all isolated aortic valve interventions were performed using catheter-based TAVI procedures. While we can only present in-hospital data here, long-term data for Germany are also available: Since 2010, the German Aortic Valve Registry (GARY) scientifically evaluated both procedures. It is organized by 2 cardiology and cardiac surgery societies (the German Society for Thoracic, Cardiac, and Vascular Surgery, DGTHG, and the German Society for Cardiology, DGK). This voluntary registry collects data from many German hospitals of patients with aortic valve disease of all kinds. Additionally, patients are informed about follow-up—with consent, longitudinal data can be collected for up to 5 years. Analysis of these data has shown a survival advantage for SAVR patients compared to TAVI patients after 5 years.^4^ A similar survival effect was observed in the pooled analysis by Barilli et al.^5^ However, these data must be interpreted cautiously due to known issues with the GARY registry (lack of completeness, as not all hospitals contribute data, some hospitals do not provide full data sets, as well as incomplete follow-up). This caution is also warranted when considering TAVI procedures for younger patients (<65 years) due to the aforementioned registry data and the as-yet unclear 10-year outcomes of TAVI valves.

Secondly, the trends in numbers of the 2 therapeutic procedures, SAVR and TAVI, over time are different: while the median EuroSCORE II and the median AKL-Kath score for TAVI patients steadily declined between 2015 and 2019, the EuroSCORE II and the AKL-Chir score for SAVR patients remained unchanged (**Figures 1-3**). The increase in risk in both the groups in 2020 is likely due to a special effect of the SARS-CoV-19 pandemic. This effect could be explained by the significant reduction in cardiology and cardiac surgery capacities during the pandemic,^6^ leading to fewer elective and lower-risk procedures while increasing the number of urgent and high-risk procedures. Another explanation could be the increased perioperative risk caused by SARS-CoV-19-induced lung disease in many patients.

The reduction in perioperative risk over time can probably be attributed to the expanded application of TAVI in patient cohorts with moderate and low risk, in line with guideline developments.^1^ With greater consideration of these lower-risk categories, a decline in mortality risk is expected. Surprisingly, the risk associated with the SAVR procedure did not increase, even though, over time, a relatively higher number of patients with florid endocarditis and pure insufficiency were treated in this group. These patients typically have a higher perioperative risk and should have increased overall cohort mortality. This effect may only become apparent in the coming years as the proportion of SAVR procedures continues to decline.

Our results indicate a systematic overestimation of TAVI risk and an underestimation of SAVR risk by the EuroSCORE II. However, when using the AKL-Kath score for the TAVI group and the AKL-Chir score for the SAVR group, perioperative mortality is well predicted (**Figures 2 and 3**). Kofler et al^7^ demonstrated this overestimation of perioperative risk by EuroSCORE II, particularly in comparison to the STS score, for the TA-TAVI group but not for TF-TAVI. There are several comparisons^8–11^ between risk scores that can be used for preoperative risk prediction in SAVR and TAVI patients, coming to mixed results with mostly a modestly discrimination of the perioperative risk. The most often compared scores (Euroscore II and STS score) do not show a clear superiority of one score over the other. A consensus document^12^ describes the involvement of laboratory and physical indices and clinical parameters for risk assessment, but does not describe a score for this. We think that the German AV score—divided into a TAVI and a SAVR arm and therefore avoiding selection bias—would be a useful tool to estimate the risk in the 2 different patient populations.

Thirdly, our analysis of QA data from the 6-year period between 2015 and 2020 in Germany shows a continuous decline in SAVR procedures by 37.6%. During the same period, TAVI procedures increased by 37.3% (**Figure 1**). By 2020, 81% of all isolated aortic valve interventions were performed using catheter-based TAVI procedures. While we can only present in-hospital data here, long-term data for Germany are also available: Since 2010, the German Aortic Valve Registry (GARY) scientifically evaluated both procedures. It is organized by 2 cardiology and cardiac surgery societies (the German Society for Thoracic, Cardiac, and Vascular Surgery, DGTHG, and the German Society for Cardiology, DGK). This voluntary registry collects data from many German hospitals of patients with aortic valve disease of all kinds. Additionally, patients are informed about follow-up—with consent, longitudinal data can be collected for up to 5 years. Analysis of these data has shown a survival advantage for SAVR patients compared to TAVI patients after 5 years.^4^ A similar survival effect was observed in the pooled analysis by Barilli et al.^5^ However, these data must be interpreted cautiously due to known issues with the GARY registry (lack of completeness, as not all hospitals contribute data, some hospitals do not provide full data sets, as well as incomplete follow-up). This caution is also warranted when considering TAVI procedures for younger patients (<65 years) due to the aforementioned registry data and the as-yet unclear 10-year outcomes of TAVI valves.

## CONCLUSION

AKL-Chir score and AKL-Cath score estimate the mortality risk of SAVR and TAVI patients more precisely than the Euroscore II.

## Data Availability

The data underlying this article were accessed from IQTIG, Berlin, Germany. The derived data generated in this research will be shared on reasonable request to the corresponding author.
